# Assessment of Interspecies Differences in Drug-Induced QTc Interval Prolongation in Cynomolgus Monkeys, Dogs and Humans

**DOI:** 10.1007/s11095-015-1760-9

**Published:** 2015-11-09

**Authors:** V. F. S. Dubois, W. E. A. de Witte, S. A. G. Visser, M. Danhof, O. Della Pasqua, on behalf of the Cardiovascular Safety Project Team, TI Pharma PKPD Platform

**Affiliations:** Leiden Academic Centre for Drug Research, Division of Pharmacology, Leiden University, Leiden, The Netherlands; Global DMPK, AstraZeneca R&D, Sodertalje, Sweden; Clinical Pharmacology Modelling & Simulation, GlaxoSmithKline, Stockley Park, Uxbridge, UK; Clinical Pharmacology & Therapeutics, University College London, London, UK

**Keywords:** cardiovascular safety, drug development, interspecies differences, PKPD modelling, QT interval prolongation

## Abstract

**Background and Purpose:**

The selection of the most suitable animal species and subsequent translation of the concentration-effect relationship to humans are critical steps for accurate assessment of the pro-arrhythmic risk of candidate molecules. The objective of this investigation was to assess quantitatively the differences in the QTc prolonging effects of moxifloxacin between cynomolgus monkeys, dogs and humans. The impact of interspecies differences is also illustrated for a new candidate molecule.

**Experimental Approach:**

Pharmacokinetic data and ECG recordings from pre-clinical protocols in monkeys and dogs and from a phase I trial in healthy subjects were identified for the purpose of this analysis. A previously established Bayesian model describing the combined effect of heart rate, circadian variation and drug effect on the QT interval was used to describe the pharmacokinetic-pharmacodynamic relationships. The probability of a ≥10 ms increase in QT was derived as measure of the pro-arrhythmic effect.

**Key Results:**

For moxifloxacin, the concentrations associated with a 50% probability of QT prolongation ≥10 ms (Cp_50_) varied from 20.3 to 6.4 and 2.6 μM in dogs, monkeys and humans, respectively. For NCE05, these values were 0.4 μM *vs* 2.0 μM for monkeys and humans, respectively.

**Conclusions and Implications:**

Our findings reveal significant interspecies differences in the QT-prolonging effect of moxifloxacin. In addition to the dissimilarity in pharmacokinetics across species, it is likely that differences in pharmacodynamics also play an important role. It appears that, regardless of the animal model used, a translation function is needed to predict concentration-effect relationships in humans.

## Introduction

The occurrence of pro-arrhythmic effects following administration of non-antiarrhythmic drugs remains an important cause of attrition in drug discovery and development. Currently, mitigation measures are in place which rely on the assessment of QTc prolongation as a surrogate marker for the risk of drug-induced Torsades de pointes (1, 2). Compounds that show any binding (affinity) and activity (inhibition) on hERG mediated K^+^ current in pre-clinical investigations (3, 4) are flagged and eventually discarded without clear understanding of their effects on cardiac repolarisation at therapeutic or supra-therapeutic concentrations in humans. Meanwhile, a myriad of experimental protocols *in vitro* and *in vivo* is used for screening and the evaluation of safety pharmacology, but little information is available on which species and experimental conditions bear clinical relevance. Specifically, most protocols have not been validated in terms of their sensitivity, specificity and predictive value. Moreover, limited attention is given to the underlying concentration-exposure relationships (5, 6).

Differently from traditional empirical research, in drug development pre-clinical *in vitro* and *in vivo* models are needed that are predictive of QT prolongation in humans at therapeutic concentrations. This requires not only insight into the intrinsic properties of a compound on the QTc interval *per se* but also into the magnitude of interspecies differences. Subsequently, accurate interpretation of such differences can be made by using the concentration-effect relationships of the compounds relative to the drug levels that are reached upon the administration of therapeutic doses. In this respect it is important to realise that drug-concentration-effect relationships can differ significantly between species (7, 8)

Recently Holzgrefe *et al.* studied the interspecies differences in QTc effects across a wide range of commonly used non-rodent species (9). Their analysis emphasised the electrophysiological basis of the interspecies differences in QTc. It is shown that by applying a correction factor based on the QT/RR relationships, similar changes in QTc interval are observed between species around the time of the maximum drug concentration after administration. However, no precise information on the pharmacokinetics in the species of interest was available, which precluded a meaningful analysis of the concentration-effect relationships (10).

By contrast, the PKPD correlation of drug effects on the QTc interval has been the subject of a number of investigations (11–14). It has been demonstrated that the actual value of QTc depends on multiple factors related to the drug and the biological system. Among the numerous methods developed to account for these complexities, a Bayesian model has been proposed that constitutes a basis for identification of drug concentration-effect relationships (14–16), An important feature of this model is that it enables a separation between drug-specific and biological system-specific factors (heart rate, circadian rhythm) that influence the value of the QT interval.

Given the relevance of non-human primates for the evaluation of the safety profile of biologicals, the aim of the current investigation was to assess quantitatively the interspecies differences in the QTc prolonging effects of moxifloxacin between cynomolgus monkeys, dogs and humans. This comparison is important because it has been suggested that monkeys might be the preferred species in pre-clinical investigations due to similarities to humans in terms of the magnitude of drug-induced QTc effects (3, 6, 17–19). Data from a new molecule is also presented to illustrate how the concept can be implemented prospectively during the screening phase.

## Methods

### “Retrospective” Evaluation – Reference Compound (Moxifloxacin)

#### Experimental Protocols in Cynomolgus Macaques

Blood samples and ECG data were collected in conscious telemetered cynomolgus macaques according to a 4-way cross-over design (*n* = 8). Four oral doses ranging between 0 and 90 mg/kg of moxifloxacin were tested, from which the 0 and 90 mg/kg dose group were used for the current investigation. The pharmacokinetics of moxifloxacin in cynomolgus monkeys was derived from venous blood samples taken at 1, 2, 4, 8 and 24 h post dose. Study design and parameters are presented in Table IA. The weight range of the monkeys was 2.8–6.8 kg. Studies were approved by the institutional Ethics Committee and conducted according to the ethical standards and GLP procedures.

**Table I:** Pre-clinical and clinical experimental protocol design and population characteristics for moxifloxacin (A) and NCE05 (B)

**A** - Moxifloxacin	Dog	Monkey	Healthy subjects
Number of animals/subjects	8	8	137
Gender	M	M	M: 88 (64%) F: 49 (36%)
Age [yr]	–	–	mean = 27 (18–50)
Dose [mg/kg]	0, 3, 10, 30	0, 90	0, 400
PK sampling times [h]	0, 0.5, 1, 2, 4, 8, 24, 36, 48	0, 1, 2, 4, 8, 24	−1, −0.5, −0.83, 0.25, 0.5, 1, 1.5, 2, 2.5, 3, 4, 6, 8, 10, 12, 24
PD sampling times	Every 1 min, averages over 24 h	Every 30 s, averages over 24 h	−1, −0.5, −0.83, 0.25, 0.5, 1, 1.5, 2, 2.5, 3, 4, 6, 8, 10, 12, 24
PK parameter	Plasma concentration	Plasma concentration	Plasma concentration
Vital signs	heart rate, blood pressure,	heart rate, blood pressure	heart rate
Demographic covariates	weight, sex	weight, sex	weight, sex
ECG parameters	LvPr, QT, RR, QRS	QT, RR	QT, QTc_b_, QTc_f_, RR

**B** - NCE05	Monkey	Healthy subjects
Number of animals/subjects	6	24
Gender	M	M
Age [yr]	–	–
Dose [mg]	0, 25, 40, 80	1, 4, 14, 30
PK sampling times [h]	0, 1.5, 4, 8, 24	−0.17, 0.33, 0.67, 1, 1.5, 2, 3, 4, 6, 8, 12, 16, 24, 36, 48
PD sampling times [h]	−1.17, −1, −0.83, −0.67, −0.5, 0.5, 1, 2, 3, 4, 6, 8, 12, 16, 20, 24 h	−0.17, 0.33, 0.67, 1, 1.5, 2, 3, 4, 6, 8, 12, 16, 24, 36, 48
PK parameter	Plasma concentration	Plasma concentration
Vital signs	heart rate	–
Demographic covariates	–	weight
ECG parameters	QT	QT, RR

*LvPr* left ventricular pressure, *QTcb* Bazett's corrected QT interval, *QTcf* Fridericia’s corrected QT interval

#### Experimental Protocols in Dogs and Humans

Details of the experimental protocols for the characterisation of the pro-arrhythmic effects of moxifloxacin in dogs and healthy subjects have been published previously elsewhere (14, 15). Briefly, clinical data for moxifloxacin were available from the positive control arm of a two-way crossover, single-blind, randomized, placebo-controlled Phase I trial in 137 healthy volunteers who received either placebo or a 400 mg dose. A summary of the relevant information for the purpose of our investigations is presented in Table I.

#### Bioanalysis of Samples from Dogs, Monkeys and Healthy Volunteers

The determination of moxifloxacin serum concentrations (Ryan Scientific, Mt. Pleasant, NC, USA) in monkeys was performed according to the methods described earlier by *Watson et al.* (19). Details on the bioanalysis of moxifloxacin can be found in *Chain and Dubois et al.* (14)

### “Prospective” Evaluation - Candidate Molecule (NCE05)

#### Experimental Protocol in Cynomolgus Macaques

Blood samples and ECG data were collected in conscious telemetered cynomolgus macaques. Six monkeys (3 female, 3 male, 2.8–6.8 kg) were treated with four oral doses: 0, 25, 40 and 80 mg/kg, of NCE05 from which the first 3 doses were used in the current investigation. Blood samples for pharmacokinetic analysis were collected at 1.5, 4, 8 and 24 h post dose.

#### Experimental Protocol in Humans

This was a Phase I, first-time-in-human, randomised, double-blind, placebo-controlled, parallel group, single centre study to assess the safety, tolerability, pharmacokinetics and pharmacodynamic effects of NCE05 following single ascending doses to healthy volunteers. Four treatment groups were tested with doses of 1, 4, 14 or 30 mg. Study design and population details can be found in Table IB. The study has been conducted in full conformance with the principles of the Declaration of Helsinki and with the local laws and regulations concerning clinical trials. The protocol and the informed consent documents have been formally approved by the relevant research Ethics Committees.

#### Bioanalysis of Samples from Monkeys

Approximately 400 μL blood was collected at each sampling occasion in lithium heparinised tubes (Microtainer®, Becton Dickinsson and CO, USA), and cooled on ice. Plasma was prepared within 30 min by centrifugation (3200 g for 5 min at +4°C). The plasma was transferred to 1.5 ml MTP system Topaz PP vial Scantec Lab and immediately frozen at −70°C until analysis.

#### Bioanalysis of Samples from Healthy Volunteers

Blood samples were collected from the forearm using vacutainer tubes with K_2_ EDTA. The blood samples were centrifuged within 30 min from collection for 10 min at 4°C. The plasma was then frozen at −20°C until analysis. Liquid chromatography and electro-spray tandem mass spectrometry (LC-MS/MS) were used to determine the total concentration of the compound in plasma.

### ECG Recordings

#### Pre-clinical Species

Pre-clinical pharmacodynamic data were collected in conscious telemetered animals. Study design and parameters are summarized in Table IA. ECG recordings were obtained by implanted radio telemetry devices. Standard aseptic surgical techniques were used for all implantation procedures. Analysis of the captured data was made via “Po-Ne-Mah” V4.1 software, and/or EMKA “ECG-Auto” version 2.4.0.30.

#### Clinical Protocol

ECG monitoring was performed with 12-lead electrocardiogram, using Marquette ECG machines measuring QT, RR, and HR. Subjects were kept in a supine position while ECG recordings were made. Details about clinical study design and populations characteristics can be found in Table IB.

### Data Analysis

#### Pharmacokinetic (PK) Data

Pharmacokinetic modelling and deconvolution techniques were used for imputation and interpolation of concentrations at the ECG assessment times. In brief, analysis of the pharmacokinetic data from the clinical and *in vivo* dog study with moxifloxacin was performed using non-linear mixed effects modelling techniques in NONMEM 6.0 and 7.1.2 (ICON, Maryland, USA), respectively. By contrast, a deconvolution method (WINNONLIN v4.1, Pharsight, USA) was used for the estimation of individual concentrations for NCE05 and moxifloxacin in cynomolgus monkeys. This method was used as no satisfactory individual prediction results could be obtained by modelling of the data in this species. The use of deconvolution is considered an acceptable approach for interpolation of the concentration data at time points between the available blood sampling times (20).

It should be noted that for the purpose of the current analysis, samples below the lower limit of quantification were set to zero. In addition, to prevent numerical difficulties during parameter estimation, all drug concentrations of the 1 mg study group for NCE05 were also set to zero. Model characteristics and parameters describing moxifloxacin pharmacokinetics are summarised in Table II.

**Table II:** Moxifloxacin pharmacokinetic model characteristics

Moxifloxacin	Dogs	Monkeys	Healthy subjects
PK model diagram		N/A	
Parameters	Ka, Vc, CL, F*	N/A	D1, Ka, CL, Vc, Vp, Q
BSV	CL		D1, Ka, CL, Vc, Vp, Q
Covariates	N/A		N/A
Derived PK time points	1–2 h: every 2 min, 2–10 h: every 5 mins 10–47 h every 15 mins	time–matched samples	1–2 h: every 2 min, 2–10 h: every 5 mins 10–47 h every 15 mins

*BSV* between-subject variability, *CL* apparent oral clearance, *D1* parameter describing zero-order absorption duration process, *Ka* absorption rate constant, *Q* intercompartmental clearance, *Vc* volume of distribution of the central compartment, *Vp* volume of distribution of the peripheral compartment

*F bioavailability, estimated separately per dose level

#### Pharmacokinetic-Pharmacodynamic (PKPD) Modelling

Model building was performed in WinBUGS version 1.4.2, as previously described in *Chain and Dubois et al.* (14, 16). In brief, the PKPD model comprises three components, which are estimated simultaneously during the fitting procedures: an individual correction factor for RR-interval, an oscillatory function describing the circadian variation of the baseline QTc values and a linear function to capture the concentration-effect relationship (15). These components are summarised in Eq. 1:

1 $$ QT=Q{T}_{C0}\cdot R{R}^{\alpha }+A\cdot \cos \left(\frac{2\pi }{24}\left(t-\phi \right)\right)+ slope\cdot C $$

where QTc_0_ [ms] is the individually corrected baseline QTc, RR [ms] is the interval between successive R waves, α is the individual heart rate correction factor, A [ms] is the amplitude of circadian rhythm, t is the clock time, φ is the phase, slope [ms/nM] is the linear concentration-effect relationship, and C is the concentration of drug at the time of QT measurements.

This type of parameterisation allows one to distinguish between system- and drug specific properties. Consequently, these parameters can be used to compare drug properties across species without the need for further correction factors. While the same model was used for the description of QT-intervals for both compounds and both species, each analysis was conducted independently. Since ECG recordings in dogs and monkeys were performed in a continuous manner, data filtering was applied to ensure data sets of workable sizes were used in the joint analysis with clinical data. Data filtering was performed taking into account the absorption and disposition profiles for the different species, so that absorption, peak and elimination phases were accurately and equally represented in a balanced dataset.

The R package R2WinBUGS was used to execute WinBUGS whilst running a session in R 2.12.8 (21). Convergence was assessed visually by monitoring the dynamic traces of Gibbs iterations and by computing the Gelman-Rubin, Geweke, Raftery-Lewis and Heidelberger-Welch test statistics for all model parameters (22–24). All PKPD parameter estimates were obtained as posterior distributions.

#### Probability of QT Interval Prolongation

A threshold of 10 ms QTc prolongation at pre-defined concentrations was selected as a measure for comparison of drug effect across species. The magnitude of this threshold was based on the assumption that if differences in sensitivity exist between species, one can easily interpret the potential implications of pro-arrhythmic activity in humans. The analysis was performed using the slope and the inter-individual correction factor for gender together with a step function in WinBUGS 1.4 (see Eq. 2) at arbitrary concentration values in such a way that data points cover the complete sigmoid curve.

2 $$ P\ge 10\;m\operatorname{s}\left( at\kern0.5em C\right)= step\left({0.00001}^{F(Gender)}\cdot slope\cdot \frac{10\;m\operatorname{s}}{C}\right) $$

where 0.00001 is set as an arbitrary small number to avoid computational errors, 10 ms is the QT interval prolongation threshold of interest, C is the drug concentration, and slope is the QT increase per unit drug concentration.

Probability curves were plotted for each compound. Subsequently, the concentration at which the probability of QT prolongation ≥10 ms is 0.5 (Cp_50_) was determined by linear regression. The safety margin, as defined by the ratio between Cp_50_ and peak concentration (Cp_50_/C_max_), was then estimated for each compound.

## Results

### Pharmacokinetics

**Fig. 1:**
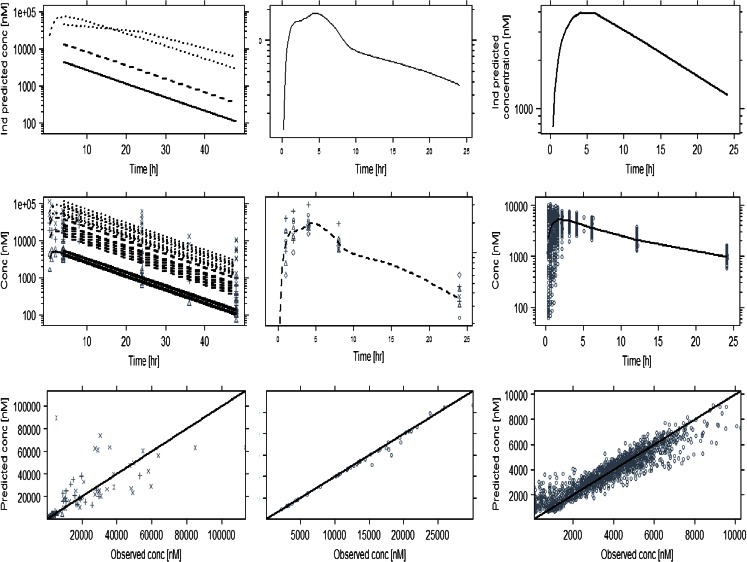
Predicted and observed pharmacokinetic profiles of moxifloxacin after administration of different dose levels to dogs (*left panels*), monkeys (*mid panels*) and humans (*right panels*). The *upper panels* show examples of the individually predicted concentrations. Mid panels: observed (*symbols*) and population predicted (*lines*) concentrations. *Lower panels*: goodness-of-fit plots depicting the observed *vs* predicted concentrations (*symbols*). The *solid line* represents the identity line

**Fig. 2:**
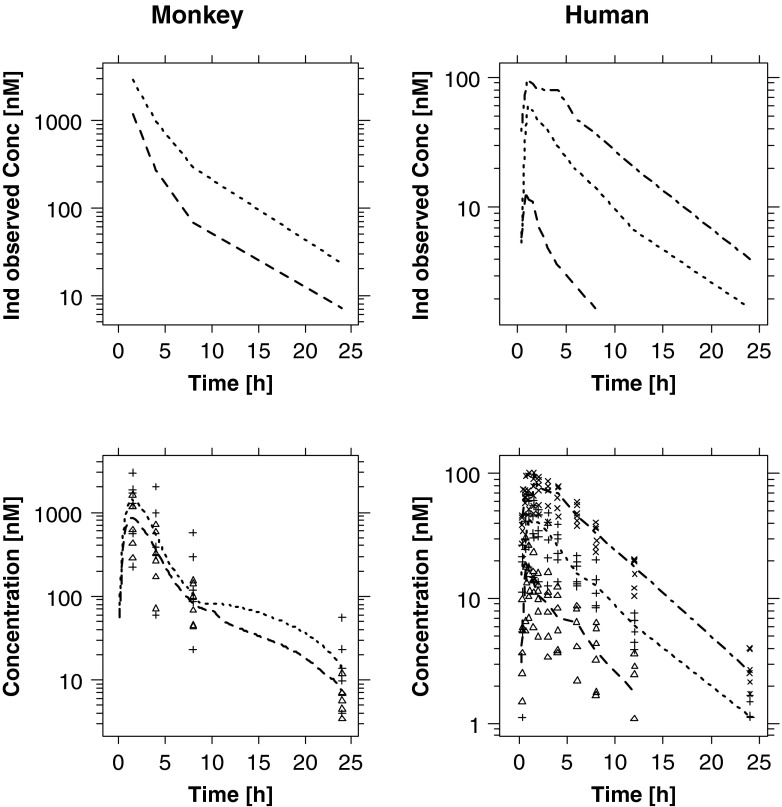
(*Upper panels*) Examples of individually predicted concentrations of NCE05. *Lower panels* depict the observed (*symbols*) and mean predicted (*lines*) concentrations in monkeys and humans. Pre-clinical doses ranged from 25 mg/kg (*dashed line*/△) to 40 mg/kg (*dotted line*/+), whereas healthy subjects received doses of 4 mg (*dashed line*/△), 14 mg (*dotted line*/+) or 30 mg (*dash dotted line*/×)

As described previously in the methods, drug concentrations at the time of ECG measurement were imputed or interpolated either by population pharmacokinetic modelling or deconvolution, depending on which technique provided the best description of the data, minimising the impact of uncertainty and poor precision in individual predicted concentrations on the PKPD analysis. Observed (time-matched), interpolated or otherwise predicted concentrations were derived at the time of the QT measurements and subsequently used for the PKPD analysis. The pharmacokinetic parameter estimates for moxifloxacin are summarised in Table III. Model predictions and goodness-of-fit plots are depicted in Figs. 1 and 2 for moxifloxacin and NCE05, respectively.

**Table III:** Moxifloxacin pharmacokinetic population parameter estimates

	Dogs		Healthy subjects	
Parameter	mean	IIV%	mean	IIV%
Ka [/h]	1.78	–	2.21	88.65
D1 [h]	–	–	0.629	85.14
CL [L/h]	3.39	21	13.4	12.69
Vc [L]	43.23	–	122	28.77
Vp [L]	–	–	55.4	44.27
Q [L/h]	–	–	78.4	–

### PKPD Modelling

**Fig. 3:**
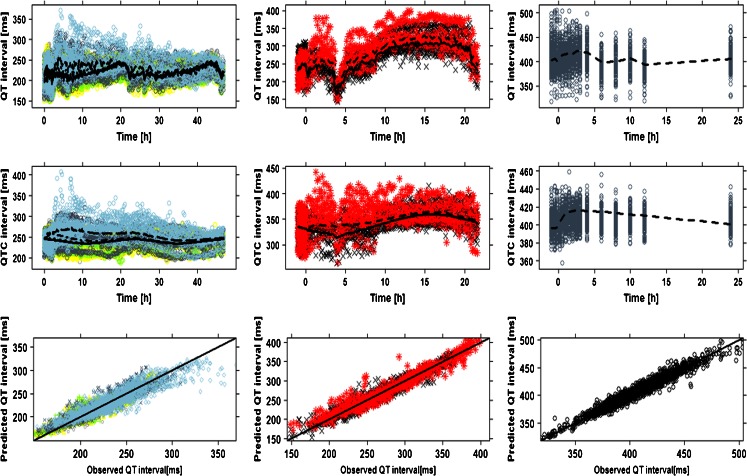
Model performance and predicted QT profiles after administration of placebo and different doses of moxifloxacin to dogs (*left panels*), cynomolgus monkeys (*mid panels*) and humans (*right panels*). Observations are indicated by *symbols*, population predictions by lines. The *solid line* in the *lower panels* represents the identity line. In dogs: ○ (*grey*) and ^_____^ are pre–dose values; △ (*yellow*) and ^_ _ _ _^ are placebo; + (*greenish*) and - - - - 3 mg/kg, x(*slate grey*) and - ^_^ - ^_^ 10 mg; ◊(*light blue*) and ^__ __ __^ 30 mg. In monkeys: × (*black*)/*solid line* are placebo; * (*red*)/*dashed line* 90 mg/kg. In humans: *dashed line* and *symbols* depict effects of a 400 mg dose

**Fig. 4:**
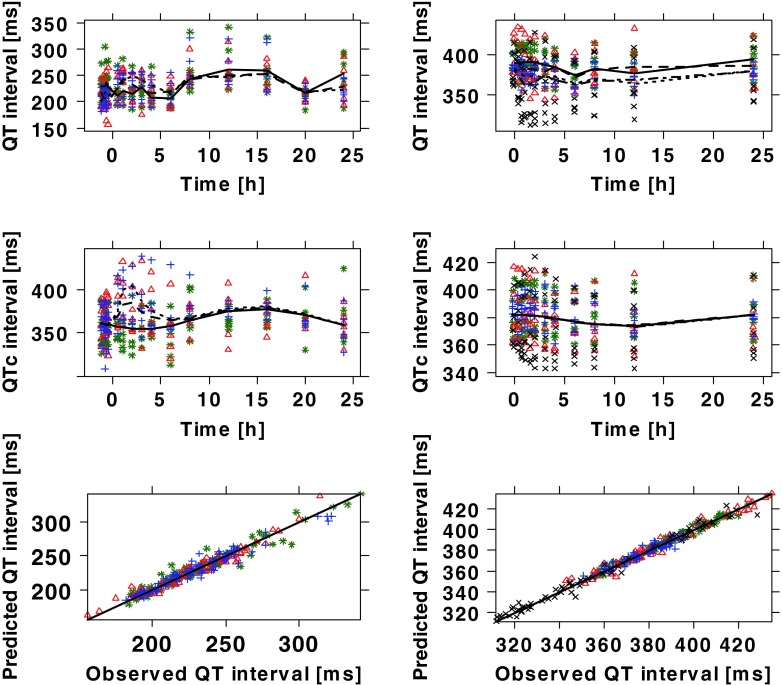
Model performance and predicted QT profiles after administration of placebo and different doses of NCE05 to cynomolgus monkeys (*left panels*) and healthy subjects (*right panels*). Observations are indicated by *symbols*, population predictions by lines. The *solid line* in the *lower panels* represents the identity line. In monkeys: * (*green*)/*solid line* are placebo; △ (*red*)/*dashed line* 25 mg/kg; + (*blue*)/*dotted line* 40 mg/kg. In humans: * (*green*)/*solid line* 1 mg; △ (*red*)/*dashed line* 4 mg; + (*blue*)/*dotted line* 14 mg; × (*black*)/*dashed-dotted line* 30 mg

The recorded ECG measurements were used for modelling purposes in conjunction with the predicted (or interpolated) drug concentrations. Diagnostic criteria and goodness-of-fit plots showed comparable model performance in all three species (Figs. 3 and 4). System specific parameters (baseline QTc (QTc_0_), QT-RR exponential correction factor (α), amplitude (A) and phase (Ф)) showed values within the same range for both drugs within dogs and monkeys. The main difference between species and compounds was found in the drug specific parameter (i.e., slope) (Table IV). The QT-RR correction factor (α) obtained for moxifloxacin and NCE05 varied significantly in humans. On the other hand, in contrast to moxifloxacin, the slope of NCE05 was not significantly different from zero, nor was there an increase in QT across the observed concentration range.

**Table IV:** PKPD parameter estimates and 95% credible intervals obtained for moxifloxacin and NCE05

	Moxifloxacin			NCE05	
	Dogs (*n* = 8)	Monkeys (*n* = 8)	Healthy subjects (*n* = 24)	Monkeys (*n* = 6)	Healthy subjects (*n* = 24)
QTc_0_ [ms]	240 (238–242)	341 (337–347)	399 (394–403)	367 (349–390)	378 (373–383)
Sex effect [ms]	N/A	N/A	8 (5–12)	N/A	N/A
α	0.28 (0.22–0.35)	0.48 (0.36–0.64)	0.40 (0.38–0.42)	0.55 (0.50–0.60)	0.26 (0.23–0.31)
A [ms]	4.6 (3.1–7.0)	14.9 (10.1–22.3)	2.4 (1.7–2.9)	7.6 (2.6–21)	4.2 (2.6–5.8)
φ [h]	23.1 (15.1–34.6)	24.9 (20.6–30.2)	10.0 (7.3–12.9)	24 (20–29)	8.1 (5.2–11)
Slope [ms/nM]	0.00056 (0.00002–0.0014)	0.0016 (0.0008–0.0026)	0.0039 (0.0033–0.0044)	0.022 (0.0097–0.041)	0.0052 (−0.0048–0.016)
BSV (QT_0_) %	6.46 (6.43–6.48)	5.41 (5.37–5.45)	5.01 (4.98–5.04)	5.2 (5.1–5.2)	5.2 (5.1–5.2)
BSV (α) %	86 (48–177)	51 (27–110)	41 (33–52)	1.9 (1.0–4.4)	19 (13–29)
BSV (A) %	9.8 (7.4–14.8)	10.4 (8.1–15.2)	5.3 (3.6–8.0)	2.6 (1.6–5.2)	8.2 (5.0–14)
BSV (φ) %	13 (8–23)	6.2 (4.1–10.3)	18 (10–31)	3.2 (1.6–7.9)	16 (8.1–34)
BSV (Slope) %	25 (17–39)	36 (25–58)	41 (29–53)	42 (23–99)	37 (21–87)
Residual Error [ms]	9.4 (9.3–9.5)	10.0 (9.8–10.3)	5.3 (5.2–5.4)	7.4 (6.8–8.1)	16 (13–19)
Prob ≥10 ms at Cmax	1.0	1.0	1.0	1.0	0
Cmax [nM]	112,930	31,400	10,300	8660	101

### Interspecies Comparison

The use of animal models for the prediction of drug effects in humans requires the identification of system specific properties. Therefore, the choice of model parameterisation is critical, in that it should allow the distinction between system-specific parameters, and drug-specific parameters and their respective variability. Despite the limited evidence from two compounds, our analysis shows similar estimates of the system-specific physiological parameters in each species. By contrast, the values of these parameters differed significantly between the species.

**Fig. 5:**
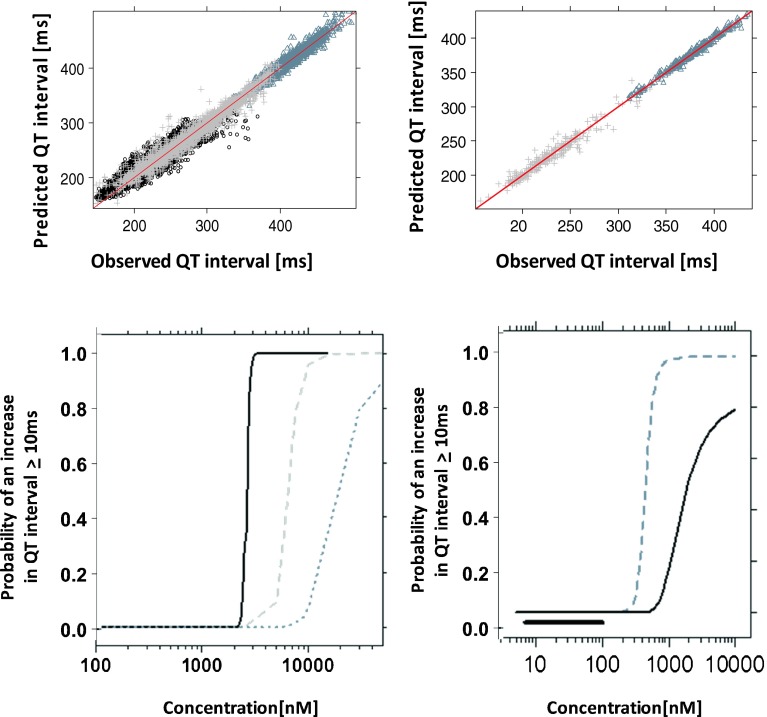
(*Upper panel*) Observed *vs* model predicted QT interval for moxifloxacin (*left*) and NCE05 (*right*). *Black circles*, *grey crosses* and *slate grey triangles* represent the experimental observations in dogs, cynomolgus monkeys and humans, respectively. (*Lower panel*) Comparison of the risk of drug-induced QTc prolongation across species. *Dotted line*: calculated values for conscious dogs; *dashed grey line*: calculated values for monkeys; *solid, black line*: calculated values for humans. The *thick black line* indicates the observed Cmax range of the clinical study

The availability of a common model to describe drug effects across species in a parametric manner offers important advantages from a drug development perspective. Figure 5 summarises the goodness-of-fit plots and the curves describing the relation between the drug concentration and the probability of a QT interval prolongation ≥10 ms. As it can be seen from the three probability curves for moxifloxacin, there is a clear shift in the PKPD relationship between preclinical species and humans, with lower values of the Cp_50_ in dogs and monkeys. In addition, it appears that the slope (i.e., the drug specific parameter) of the relationship between drug concentration and QT interval prolongation also differs between the species for each drug.

Our attempt to illustrate the use of the approach for prospective evaluation of novel compounds showed that the magnitude of the changes in parameter estimates may not be systematic, i.e., a higher slope parameter was observed in monkeys following administration of NCE05, as compared to humans. The Cp_50_ values derived from the probability curves were 6469 *vs* 2597 nM for moxifloxacin and 449 *vs* 2910 nM for NCE05 in monkeys and humans, respectively. Based on these estimates, safety margins (Cp_50_/C_max_) were found to be 0.62 *vs* 0.25 for moxifloxacin and 4.5 *vs* 29.1 for NCE05 in monkeys and humans, respectively.

## Discussion and Conclusions

Our findings show that a model-based approach can be used to quantitatively assess interspecies differences in the QT prolonging effect of moxifloxacin, confirming the feasibility of the approach in a second non-clinical species after our first results comparing drug effects in dogs and humans. From a drug development perspective, the availability of a general PKPD model enables the evaluation of the pharmacological (QT interval prolonging) effect in humans, taking into account clinically relevant exposure ranges. Using a reference compound and novel candidate molecule and explicit distinction between drug-specific and system-specific parameters, our results suggest that intrinsic differences exist in the PKPD relationships in dogs and monkeys, which make the direct extrapolation or translation of drug effects from these pre-clinical animal species humans rather challenging.

### PKPD Modelling

Despite the increased understanding of PKPD relationships and the role of modelling and simulation in drug development, prospective use of PKPD models as a predictive tool for the evaluation of drug safety has been limited (25). This is partly explained by model building requirements, which are often time-consuming and impose appropriate experimental design and dose rationale. One important feature of our analysis was therefore to illustrate how PKPD relationships in pre-clinical species can be used to identify potential differences between animals and humans.

As shown in Fig. 5, the modular structure of the model, including systems- and drug-specific parameters allowed direct comparison of drug-induced effects across species. In fact, our results for moxifloxacin indicate that drug-induced QT prolonging effects occur at different exposure ranges in dogs, monkeys and humans. These data can be integrated, as illustrated here, to estimate the probability of QT interval prolongation ≥10 ms. Contrary to prevailing views about the sensitivity of pre-clinical species to detect safety signals, the probability curve in humans clearly shows a steeper increase across the therapeutic concentration range of each compound, indicating the risk of QT prolongation ≥10 ms at such levels. In contrast, the maximum probability of QT prolongation occurs at higher exposure in dogs and monkeys than in humans, suggesting differences in the sensitivity to the QT-prolonging effects across species.

### Experimental Protocols for Drug Screening

Despite the wide number of experimental protocols for *in vivo* screening, uncertainty exists about which species and experimental conditions bear clinical relevance and hence can be used as basis for decision-making. Clearly, any attempt to translate pre-clinical findings must account for the impact of experimental procedures on the measures and parameters of interest (26). Consequently, the lack of attention to clinically relevant aspects in safety pharmacology experiments is probably one of the major hurdles in identifying interspecies correlations or scaling functions.

The first element worth mentioning is the target exposure range in preclinical protocols and the expected therapeutic and supra-therapeutic levels in humans. Clearly, interspecies differences in drug disposition, metabolism and bioavailability need to be factored in to ensure appropriate levels are reached and to enable an unambiguous interpretation of the results.

The available protocols rely on empirical choices for the dose selection. For instance, moxifloxacin peak concentrations differed only three fold between monkeys and humans, whilst an 86-fold difference was observed for the new molecule NCE05. Such a difference in exposure impairs the comparison of PKPD relationships, as overexposure of such a magnitude may lead to other adverse events, which in turn can mask the primary pharmacological (QT prolonging) effects. In addition, multiple interactions may occur, with potentially antagonising effects if the compound under investigation shows affinity for more than one specific ion channel (27, 28). Extrinsic sources of variability may also contribute to noise. Food intake (usually at 4 h post dose) in pre-clinical protocols can greatly affect QT interval, which will decrease irrespective of drug levels, as can be seen in the moxifloxacin data (Fig. 3). The correction for RR differences does reduce most of the food-induced QT decrease, but residual differences may mask the intrinsic effects of candidate molecules which cause small prolongation (29, 30).

Another important design aspect is the use of informative sampling schemes, with sampling intervals and frequencies that allow correct characterisation of the pharmacokinetics. For instance, the standard experimental design used for NCE05 had important limitations, as samples were lacking in the first 1.5 h post dose, a period during which the absorption process occurs and where consequently the highest peak concentrations might have occurred. Absence of such information impairs the estimation of peak levels and possibly maximum drug effects.

### Translational Pharmacology

As a comparison of the parameter estimates with published literature data is not possible, it appears from the present investigation that dogs are less sensitive than monkeys and monkeys slightly less sensitive than humans to the QT-prolonging effects of moxifloxacin. This can be seen by the difference in the slope parameter of the concentration-effect relationship and the difference in the probability of reaching ≥10 ms increase in QTc interval at exposure levels corresponding to the approved therapeutic doses. More specifically, for moxifloxacin the differences in the slopes of the linear concentration-effect relations between monkeys (0.0016 ms/nM) and humans (0.0039 ms/nM) is smaller than in dogs (0.00056) (14). As these results are all obtained with the same model, they can be compared reliably.

On the other hand, it is well known that concentration-effect relationships can differ between species, as result of for example differences in homeostatic processes, circadian variation, target expression and/or transduction mechanisms (31–34). Such differences need to be taken into account in the extrapolation of drug effects from pre-clinical species to humans, possibly by means of a system-specific translation function (34–36). In fact, the topic of interspecies scaling of drug-induced QTc interval prolongation was recently addressed in an extensive investigation by *Holzgrefe et al.* (9). Their work however has not considered the role of pharmacokinetics and therefore a detailed and meaningful analysis of the PKPD relationship over a wide concentration range was not possible. Specifically, a concentration-effect correlation was estimated by assuming a dose scaling factor and using the data obtained in a 1-h time interval around the Tmax, i.e., an empirical approach which disregards the differences in the pharmacokinetic profile of different compounds (14, 37).

From a drug development perspective our approach offers the opportunity to use modelling and simulation for the prospective evaluation of novel compounds (38). By contrast, recent examples of the analysis of QTc interval data fail to create a direct, quantitative correlation between clinical data and pre-clinical findings. In cases where this was attempted, additional *in vitro* data was needed in order to identify a plausible correlation between species (13, 39). A limiting factor in these analyses is that the model cannot be reused for any other compound or compound class. This rather undermines the intended utility of such models; namely to support the screening of compounds in the early stages of drug development.

#### Limitations

We have made assumptions about the data sets available from the trials and experimental protocols. Firstly, we have assumed that monkey data from the top dose yielded information on the clinically relevant exposure levels of moxifloxacin, whereas the placebo arm was sufficient to capture the natural variation in baseline and system-specific parameters. The data from intermediate dose levels was found to be unsuitable for modelling purposes.

In the data available from the clinical trial with NCE05, details on baseline assessment day were missing. However, a very low dose was administered (1 mg) to healthy subjects in one of the arms, which resulted in very low concentrations, close to the LLOQ. In order to strengthen the estimation of the system specific parameters, it was decided to set the drug concentrations to zero for all assessment times and treat this arm as the baseline (placebo) data.

We have also made assumptions about the low probability of describing drug effects based on a traditional Emax model, given that a hyperbolic function may not be observable *in vivo* due to arrhythmias in pre-clinical species and in humans. In addition, in clinical trials subjects are withdrawn from the trials if ECG stopping criteria are reached (e.g. QTc > 500 ms). Considering these factors, estimation of a maximum effect may be difficult and a small misspecification of this parameter could lead to a considerable bias in the estimation of the potency parameter, IC_50_. As a 10 ms increase in the QT interval can increase the risk on TdP significantly, bias in IC_50_ estimates can lead to false positive or false negative results and wrong conclusions about the pro-arrhythmic risk. A linear concentration-effect relationship was therefore deemed to reflect the lower end of the ascending portion of a theoretical Emax curve. It should be noted that whilst the use of the Emax model has been shown to result in some improvement in terms of the precision of parameter estimates and residual variability (12, 40, 41), other examples are also available where a linear effect model showed better performance (42, 43).

Another important point is the assumption that NCE05 shares the same pro-arrhythmic mechanisms at low and high exposure levels. The exclusion of the high dose level from the analysis was based on the clinical relevance of concentration range observed in the lower dose groups. Furthermore, it should be noted that one cannot exclude the role of sample size as a determinant of the wide credible intervals for drug-specific parameters Uncertainty in parameter estimates may be partly explained by the limited data available in the therapeutic concentration range.

Quantitative data are lacking to allow a somewhat mechanistic explanation of the observed difference in drug sensitivities and system specific parameters in dogs and monkeys. Although our model can be used to characterise interspecies differences in drug sensitivity, the contribution of multiple underlying mechanism of QTc prolongation should not be overlooked. Compounds with variable degree of affinity for different ion channels will behave differently across species depending on the expression level and overall tissue density of the each channel (sub) type.

Lastly, we should emphasise the implications of poor quality data on drug disposition properties. Inadequate dose rationale or sub-optimal blood sampling schemes can lead to model misspecification, bias and poor precision in PK and PKPD parameter estimates. Such poor experimental conditions will have direct impact on the drug-specific parameter (i.e., the slope in Eq. 1), defeating the objectives of quantitative PKPD modelling.

In summary, the use of a model-based approach has enabled the assessment of the interspecies differences in the concentration-QT prolonging effect relationship of moxifloxacin. These differences are reflected by the probability threshold for QT interval prolongation ≥10 ms, which was found to differ between species. Irrespective of the intrinsic differences in cardiac function between dogs, monkeys and humans, our approach shows that accurate evaluation of the pro-arrhythmic potential of a novel compound cannot be performed without disentangling drug-specific parameters from system-specific parameters.

The current results also suggest that different experimental protocols may be required, including potentially larger sample sizes in dogs as monkeys to allow estimation of model parameters with sufficient precision. The observed lower sensitivity of dogs to the effects of moxifloxacin, as compared to monkeys has been suggested previously and seems to be supported by this initial analysis. Further investigation using multiple compounds with known arrhythmogenic properties is needed to assess whether the observed differences across species are systematic.

## Abbreviations

Cmax: Peak drug concentration
Cp_50_: Concentration associated with a 50% probability of QT interval prolongation ≥10 ms
ECG: Electrocardiogram
EDTA: Ethylenediaminetetraacetic acid
GLP: Good laboratory practice
hERG: Human Ether-à-go-go-Related Gene
HR: Heart rate
LOQ: Limit of quantification
PD: Pharmacodynamic(s)
PK: Pharmacokinetic(s)
PKPD: Pharmacokinetic-pharmacodynamic
QTc: Heart rate corrected QT interval
